# Lignin biosynthesis and accumulation in response to abiotic stresses in woody plants

**DOI:** 10.48130/FR-2022-0009

**Published:** 2022-07-12

**Authors:** Xiaojiao Han, Yanqiu Zhao, Yinjie Chen, Jing Xu, Cheng Jiang, Xiaqin Wang, Renying Zhuo, Meng-Zhu Lu, Jin Zhang

**Affiliations:** 1 State Key Laboratory of Tree Genetics and Breeding, Key Laboratory of Tree Breeding of Zhejiang Province, Research Institute of Subtropical Forestry, Chinese Academy of Forestry, Hangzhou, Zhejiang 311400, China; 2 State Key Laboratory of Subtropical Silviculture, College of Forestry and Biotechnology, Zhejiang A & F University, Hangzhou, Zhejiang 311300, China

**Keywords:** Woody plants, Global climate change, Abiotic stresses, Lignin, Transcription factors

## Abstract

Woody plants have to experience various abiotic stresses due to their immobility and perennial characteristics. However, woody plants have evolved a series of specific regulation pathways in physiological and molecular mechanisms to deal with adverse environments. Compared with herbaceous plants, perennial woody plants have the advantages of developed roots and hard stems, and increased secondary xylem, which can strengthen the vascular system of the plants. The lignification process involves the lignin deposition on the cell wall by oxidation and polymerization of lignin monomer, which plays an important role in abiotic stress tolerance. This review focuses on recent progress in the biosynthesis, content, and accumulation of lignin in response to various abiotic stresses in plants. The role of transcription factors is also discussed in regulating lignin biosynthesis to enhance abiotic stress tolerance *via* changing cell wall lignification. Although woody plants shared similar lignin biosynthesis mechanisms with herbaceous plants, the temporal and spatial expression and stress response profiles of lignin biosynthetic genes provide the basis for the differences in stress tolerance of various species. An in-depth understanding of the role of lignin in the abiotic stress tolerance of woody plants will lay the foundation for the next step in tree resistance breeding through genetic engineering.

## Introduction

Global warming and extreme climatic events such as high temperature, drought, and floods have always been a hot topic, and have attracted the attention of scientific researchers. Climate change has brought multiple negative effects on the environment. For example, heavy rainfalls after prolonged periods of drought have caused accelerated permeation of soil moisture and accumulation of salts in the soil^[1]^. Increased carbon dioxide (CO_2_) from anthropogenic emissions, one of the most important greenhouse gases, is also attributed to global climate change^[2]^. Furthermore, heavy metal (HM) pollution caused by anthropogenic activities such as mining, smelting and application of fertilizers is increasing worldwide, and these activities lead to HMs leaching into groundwater or accumulating on the soil surface^[3]^. The above-mentioned natural disasters from climate change and HM pollution have caused serious damage to the growth, development, and reproduction of plants^[4]^. How plants (especially woody plants with long life cycles) deal with adverse environments is a fundamental biological issue that needs to be better understood. In the long-term evolutionary process, woody plants have evolved a series of physiological and molecular mechanisms, including enhanced secondary development and metabolic requirements for secondary growth, to coordinate growth and development under stress conditions. Plant cell walls consist of lignin, carbohydrate poslymers and structural proteins in variable amounts, and its function not only maintains the cell shape, but also provides mechanical strength to withstand the expansion pressure^[5]^. Among the cell wall components, lignin offers a barrier against a variety of abiotic and biotic stresses^[6]^.

As a phenolic biopolymer, lignin is one of the important products of the plant phenylpropanoid biosynthesis pathway in vascular plants. In higher plants, lignin in the plant cell walls accounts for about 30% of the total organic carbon in the biosphere. Woody trees, such as *Populus trichocarpa*, contain ~70% syringyl lignin monomers (S-lignin) and ~30% guaiacyl lignin monomers (G-lignin), with a small amount of hydroxyphenyl lignin (H-lignin)^[7]^, which have more S-lignin than herbaceous plants^[8]^. The biosynthesis of lignin monomer undergoes a series of enzymatic reactions. These reactions are catalyzed by at least 11 enzymes: phenylalanine ammonia lyase (PAL), cinnamate 4-hydroxylase (C4H), *p*-coumarate 3-hydroxylase (C3H), 4-coumarate: coenzyme A ligase (4CL), 5-hydroxyconiferyl aldehyde *O*-methyltransferase (AldOMT), coniferyl aldehyde/ferulate 5-hydroxylase (CAld5H/F5H), *p*-hydroxycinnamoyl-CoA shikimate/quinate hydroxycinnamoyl transferase (HCT), caffeoyl-CoA *O*-methyltransferase (CCoAOMT), cinnamoyl-CoA reductase (CCR), caffeic acid *O*-methyltransferase (COMT) and cinnamyl alcohol dehydrogenase (CAD), and caffeoyl shikimate esterase (CSE)^[9]^. Finally, monolignols are polymerized into lignin polymers by laccases (LACs), peroxidases (PRXs/PODs/PERs), polyphenol oxidases and coniferyl alcohol oxidases^[6]^.

Under various abiotic stresses such as salt, waterlogging, drought, temperature and HMs, plants grow abnormally and are accompanied by physiological changes^[10]^. These stresses can significantly change the lignin content or affect its composition and structural rearrangement^[11]^. In this review, we summarize the recent progress in the role of lignin in abiotic stresses in woody plants. This provides a theoretical basis for the cultivation of tree species with enhanced stress resistance by regulating lignin biosynthesis in the future.

## Structural genes of lignin biosynthesis involved in abiotic stress responses

Lignin is often deemed to be important for plant survival, since lignin content usually increases under abiotic stresses in roots and leaves (Table 1). For example, in *Eucalyptus urograndis*, drought decreased the amount of lignin in the stem apical regions and increased lignin in the basal regions of the stem, and reduced the S/G ratio (syringyl/guaiacyl lignin unit) in the basal regions^[12]^. In wax apples (*Syzygium samarangense*), lignin accumulation in the epidermis and cortex was found in older roots in both normal and flooded batches^[13]^. When *Coffea arabica* was exposed to salt stress, the lignin monomer content of the G and S units increased in the leaf cell wall, implying that the lignin composition is involved in the salt response of woody plants (Table 1)^[14]^. Hence, changes of lignin content or composition may be of great importance for adaptation to abiotic stresses in plants.

**Table 1:** Effects of different abiotic stresses on lignin in woody plants.

Stress	Species	Tissue	Treatment	Main effects	Reference
Drought	*Populus tomentosa*	*PtoMYB170*-overexpressing *Arabidopsis*	Desiccated without watering for 2 weeks and then rewatered	Promoted lignin deposition by activating *CCR2*, *COMT*,* CCoAOMT1* and *C4H2*	Xu et al.^[91]^
	*Populus trichocarpa*	4-month-old *OE-PtrbHLH186-L4* plants	Soil relative water content was reduced from 60% to 13% by withholding water	Transregulated many monolignol genes and increased in G subunits	Liu et al.^[85]^
	*Leucaena leucocephala*	Stem and root	1% mannitol (*w*/*v*)	Increased lignification and corresponding CCR protein accumulation	Srivastava et al.^[16]^
	*Eucalyptus urograndis*	Basal and apical regions of the stem	Not irrigated until wilt	Promoted lignin deposition and reduced the S/G ratio in the basal regions	Moura-Sobczak et al.^[12]^
	*Populus trichocarpa*	Young shoot tissues (top 3 cm)	Supplying 150 ml water once per week for 4 weeks	Decreased the lignin S/G ratio in young shoots	Hori et al.^[17]^
Flooding	*Populus* × *canescens*	Root	Water level of the containers exceeded the soil surface by 2 to 3 cm	Down-regulated lignin biosynthesis genes	Kreuzwieser et al.^[20]^
	*Syzygium samarangense*	Root	The flooded pots maintained a depth of several centimeters of water in a plastic dish placed under the pots	Lignin accumulation in epidermis and cortex in both normal and flooded batches	Tuladhar et al.^[13]^
Salt	*Populus canescen*s and *Populus euphratica*	Leaf, stem, root	25 or 100 mM NaCl	Increased in the lignin: carbohydrate ratio in both species.	Janz et al.^[21]^
	*Betula platyphylla*	Leaf of *BpNAC012*-overexpressing lines	200 mM NaCl	Elicited higher expression levels of lignin biosynthetic genes and elevated lignin accumulation in *BpNAC012*-overexpressing lines	Hu et al.^[79]^
	*Betula platyphylla*	*BplMYB46*-overexpressing lines	200 mM NaCl for 10 d	*BplMYB46* improved salt and osmotic tolerance by increasing lignin deposition	Guo et al.^[80]^
	*Malus × domestica*	Leaf of *MdSND1*-overexpressing and *MdSND1*-RNAi lines	200 mM NaCl	*MdSND1* is directly involved in the regulation of lignin biosynthesis.	Chen et al.^[78]^
	*Simmondsia chinensis*	Leaf	50, 100 or 200 mM NaCl	Reduced lignin production	Alghamdi et al.^[23]^
	*Coffea arabica*	Leaf	50, 100 or 150 mM NaCl	Cell walls of coffee leaves have undergone changes in the polysaccharide and lignin composition	de Lima et al.^[14]^
	*Tamarix hispida*	Root	400 mM NaCl	Two genes *SAMS* and *COMT* involved in lignin synthesis were highly responsive to NaCl stress	Li et al.^[22]^
Heat	*Eriobotrya japonica*	Fruit	40 °C	*EjHSF1* *trans*-activated the lignin biosynthesis-related genes	Zeng et al.^[94]^
Cold	*Eriobotrya japonica*	Fruit	0 or 5 °C	Chilling condition during postharvest storage lead to increased expression levels of *PAL*,* 4CL* and *CAD*, and resulted in increased lignin content	Liu et al.^[32]^
	*Populus tremula* ×* P. tremuloides* cv. Muhs1	Stem	10 °C	Increased lignin contents in cuttings	Hausman et al.^[33]^
Heavy metals	*Pinus sylvestris*	Needles, stem, and roots	0.5, 1, 2, or 4 mM Al(NO_3_)_3_	Roots affected by Al showed deformation in cell walls and higher lignification and suberization.	Oleksyn et al.^[41]^
	*Citrus sinensis* and *Citrus grandis*	Leaf, stem, root	1.0 mM AlCl_3_·6H_2_O	DCBC protein promoted the synthesis of lignin to achieve the ability to immobilize Al	Wu et al.^[45]^
	Nine *Myrtaceae* species	roots	1.0 mM AlCl_3_·6H_2_O	Lignin was formed only in the root tips in *Melaleuca bracteata*	Tahara et al.^[40]^
	*Camellia sinensis*	Root, leaf	Al_2_(SO_4_)_3_·18H_2_O	The down-regulation of F5H and the binding of Al and phenolic acids reduced the accumulation of lignin	Xu et al.^[44]^
	*Camellia sinensis*	Roots or in cultured cells	400 µM AlCl_3_	Reduced the activities of PAL and POD, and lignin content	Ghanati et al.^[43]^
	*Camellia sinensis*	Callus Cultures	106 µM Cd(NO_3_)_2_	Increased the lignin content in the root and stem calli	Zagoskina et al.^[46]^
	*Avicennia schaueriana*	Leaf, stem, root	0, 5, 15, 30, or 45 mg·L^−1^ CdCl_2_·5/2H_2_O	Induced lignin deposition in xylem cells of all vegetative organs	Garcia et al.^[47]^
	*Populus* × *canescens*	Leaf, wood, root	50 µM CdSO_4_	Increased in GH3 activities and thereby shunted the metabolism to enhanced formation of lignin.	Elobeid et al.^[48]^
	Scots pine (*Pinus sylvestris*)	Root	5 or 50 µM Cd	50 µM Cd resulted in significant increases in lignin content	Schützendübel et al.^[49]^
	Two *Salix caprea* isolates	Root	0.5 mg·L^−1^ CdNO_3_·4H_2_O	KH21 delayed the development of apoplastic barriers	Vaculík et al.^[53]^
	*Bruguiera gymnorrhiza* and *Rhizophora stylosa*	Root, leaf	100, 200, 300, or 400 mg·kg^−1^ CuCl_2_	Increased lignification in mangroves roots	Cheng et al.^[60]^
	Six species of mangroves	Root	400 µg·kg^−1^ ZnCl_2_, 200 µg·kg^−1^ CuCl_2_	Three high metal-tolerant rhizophoraceous species exhibited a thick exodermis with high lignification	Cheng et al.^[61]^
Light	*Pinus radiata*	Callus cultures	From the dark to a 16 h photoperiod	Enhanced the enzymes CAD and PAL, and increased the amount of lignin	Möller et al.^[64]^
	*Malus domestica*	Fruit	Direct sunlight, shaded, and severe sun damage	Induced the expression levels of* MdCOMT1* and *MdCAD* in the flesh, and increased the accumulation of lignin in cell walls of the flesh and skin in sudden exposure	Torres et al.^[65]^
	*Fagus sylvatica*	Leaf	Leaves of the sun and shade crown	Induced higher lignin contents in leaves in shade crown than in sun crown under ambient O_3_ concentrations	Jehnes et al.^[62]^
CO_2_	*Populus tremula* × *alba*	Stems of young tree	Elevated CO_2_ (800 µL·L^−1^)	Increased lignin content in lower and middle stems	Richet et al.^[67]^
	*Betula pendula*	Green leaves	Elevated CO_2_ (2 × ambient)	Decreased contents of acid-soluble lignin in birch leaves	Oksanen et al.^[68]^
	*Fagus sylvatica*	Two-year-old beech seedlings	Ambient and elevated CO_2_	Leaves in the elevated CO_2_ treatment contained less lignin than leaves in the ambient CO_2_ treatment	Blaschke et al.^[69]^

### Water stresses

#### Drought

Extensive literature proves that drought stress tolerance of plants is associated with the degree of lignification. Under drought stress, the expression of lignin biosynthesis-related genes, such as *CAD*, cytochrome proteins (*CYP450*), and S-adenosyl-L-methionine synthetase (*SAMS*), changed significantly in maize leaf, suggesting that lignin content is an effective indicator for evaluating drought tolerance in maize^[15]^. Similarly, the accumulation of CCR protein and enhanced cell wall lignification was observed in the stems of *Leucaena leucocephala* seedlings under mannitol-simulated drought treatment^[16]^. In contrast, the total lignin content of young shoots and mature stems of *P. trichocarpa* did not change under drought conditions, while the S/G ratio decreased significantly in young shoots and was accompanied by the down-regulation of the S-lignin biosynthetic genes *COMT* and *F5H*^[17]^. The above results indicate that woody plants shared similar lignin biosynthetic mechanisms with herbaceous plants under drought stress. Although significant progress has been made in the study of lignin content and its structural changes under drought stress in recent years, there is still a lot of work to be done in the screening of lignin-related key genes for drought tolerance and their application in tree molecular breeding.

#### Flooding

**Figure 1:**
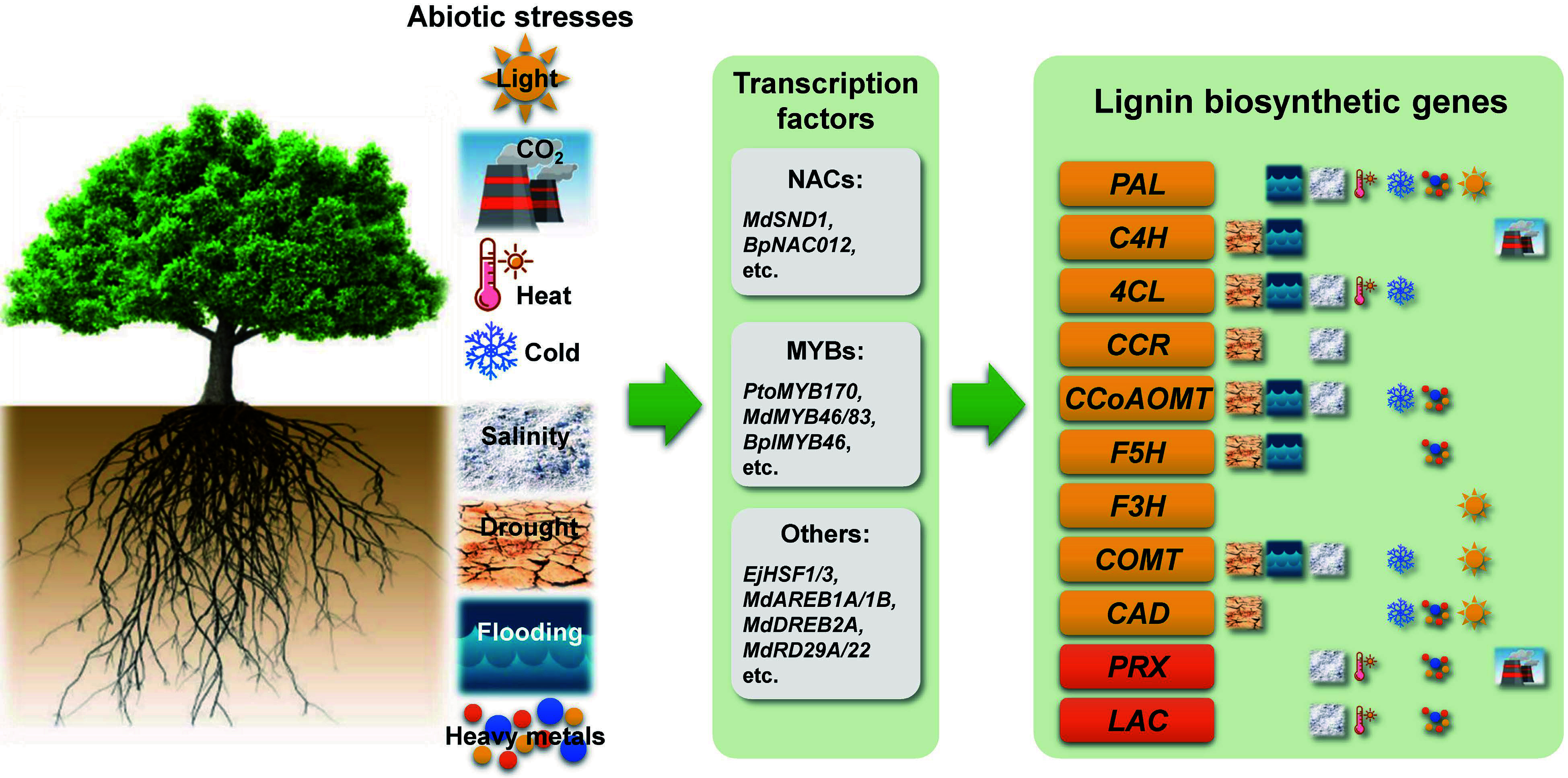
Schematic diagram summarizing the various abiotic stresses affecting lignin biosynthesis in woody plants. Phenylalanine ammonia lyase (PAL), cinnamate 4-hydroxylase (C4H), 4-coumarate:coenzyme A ligase (4CL), cinnamoyl-CoA reductase (CCR), caffeoyl-CoA *O*-methyltransferase (CCoAOMT), ferulate 5-hydroxylase (F5H), flavonoid 3-hydroxylase (F3H), caffeic acid *O*-methyltransferase (COMT), cinnamyl alcohol dehydrogenase (CAD), peroxidase (PRX), and laccase (LAC).

Flooding often occurs together with a variety of natural disasters, which make plants in a low-oxygen environment under soil waterlogging conditions, thereby affecting the growth and development of plants^[18]^. Comparative proteomics and lignin staining analysis of *Glycine max* L. revealed that flooding stress decreases cell wall proteins biosynthesis and lignin deposition in seedling roots and hypocotyls^[19]^. Molecular responses of gray poplar (*Populus* × *canescens*) following flooding suggest that lignin biosynthesis genes (*PAL*,* C4H*, *4CL*, *F5H*, *COMT*, and *CCoAOMT*) were significantly down-regulated in the root (Table 1, Fig. 1)^[20]^. In summary, lignin deposition plays an important role in the resistance of woody plants to water deficit and waterlogging.

### Salt stress

With the development of high-throughput sequencing technology, more lignin biosynthesis-related genes have been identified as participating in salt stress response. Janz et al. compared the transcriptional responses of a salt-sensitive poplar (*Populus* × *canescens*) and a salt-tolerant poplar (*P. euphratica*) to salinity, and found that genes related to flavonoid production and precursors of lignin (such as genes encoding flavonoid 3-hydroxylase, flavonol synthase, flavonol *O*-methyl transferase, cytochrome P450 family protein, peroxidases and CCR) were activated by salinity in the developing xylem^[21]^. *Tamarix* *hispida* has a strong tolerance to salt stress and is an ideal woody plant for preventing saline-alkali land degradation. The expression sequence tags were analyzed in *T.* *hispida* root treated with 400 mM NaCl at 0, 24, and 48 h, and two lignin biosynthetic genes (*S-Adenosyl-L-methionine synthase* and *COMT*) were highly induced at 24 h^[22]^. RNA-Seq analysis of jojoba (*Simmondsia chinensis*) leaves demonstrated that multiple genes related to cell wall remodeling and lignin biosynthesis such as *Xyloglucan endotransglucosylase/hydrolase protein 7* and *BURP domain protein RD22* were significantly up-regulated under salt stress^[23]^. These results indicate that enhanced lignin biosynthesis has a positive effect on the salt tolerance of woody plants (Fig. 1).

### Temperature stresses

#### High temperature

Perennial woody plants need to go through multiple annual cycles, and frequently suffer extreme temperature stresses including high and low temperatures. With the continuous warming of the global climate caused by greenhouse gas emissions, plants have evolved multiple response mechanisms that adapt to rising ambient temperatures. In strawberry, high temperature enhances the activities of active oxygen species-scavenging enzymes PRXs^[24]^, which catalyzes the phenoxy radical formations from monolignols. Physiological and transcriptome analyses of the thermo-tolerance mechanism of *Oryza sativa* L. during the reproductive stage revealed that 77 genes involved in lignin deposition were significantly differentially expressed, including 12 *LACs* and 21 *PRXs*, and more lignin was deposited in spikelet at 3 days under heat stress (Fig. 1)^[25]^. This result is consistent with observations in roots and stems of* Agrostis* grass species, which indicates that high temperature may regulate lignin biosynthesis through increasing *LAC* gene expression^[26]^. Thus, LACs may be involved in eliminating the overproduction of H_2_O_2_ during heat stress and promoting the lignin biosynthetic pathway in plant heat resistance. Furthermore, the content of G- and S-lignin increases while H-lignin decreases in coffee (*Coffea arabica L.*) leaves at 5 days under heat stress, which is very important for its adaptation under high temperature (Table 1)^[27]^. Therefore, changes in the content and composition of lignin in the cell wall have a buffering effect on the adaptability of woody plants under elevated temperature environments.

#### Low temperature

Cold stress modifies gene expression and plant metabolism, thereby affecting many biological functions^[28]^. The plant cell wall, as the mechanical support of the cell, is essential for the survival of plant cells during freezing, and its lignin deposition is directly related to freezing tolerance via enhancing the mechanical strength of root by hardening the cell wall at the root tip^[29]^. In *Arabidopsis*, the cold-induced nuclear protein Tolerant to Chilling and Freezing 1 (TCF1) interacts with histones H3 and H4, and binds to the chromatin containing *BLUE-COPPER-BINDING PROTEIN* (*BCB*) to regulate lignin biosynthesis ^[^^30]^. During cold conditions (−8 °C or −10 °C for 2 h), *TCF1* is rapidly induced to activate *BCB* transcription and then stimulates expression of *PAL1/3/4* to maintain lignin accumulation^[30]^. After 75 days of treatment at low temperature of 14.5 °C, the down-regulation of *ShCAD2*, *ShCOMT1* and *ShCCoAOMT1* in the rind of sugarcane (*Saccharum* spp.) stem resulted in a decrease in lignin content, while the up-regulation of *ShF5H* in pith increased in lignin content^[31]^. In addition, the expression of *PAL*, *4CL* and *CAD* increased during low-temperature storage of loquat fruits after harvest, leading to an increase in lignin content, which may be related to the early formation of secondary cell walls induced by low temperature (Fig. 1)^[32]^. Under field conditions, the chilling stress during the early spring growth period caused serious damage to the growth of forest trees. Under low temperature conditions of 10 °C, the lignin content in the shoot of the 3-month-old poplar (*Populus tremula × P. tremuloides* L. cv. Muhs1) seedlings increases, but this is not the case in the *in vitro* system (Table 1)^[33]^. In summary, changes in lignin content and composition can improve the elasticity of cell walls to increase the ability to adapt to the growth of ice crystals and reduce the damage to cells caused by dehydration during the cold acclimation process.

### Heavy metal stresses

#### Aluminium (Al)

HM pollution caused by the rapid development of industrialization and traditional agricultural irrigation is adversely affecting the sustainable development of the economy and causing serious damage to plants^[34]^. The cell wall is the first structure that plant cells contact with HMs, and it is also a more effective barrier against toxic metals from the external environment via increasing cell wall lignification to prevent heavy metals from entering cells under metal stress conditions^[35,36]^. Meanwhile, the cell wall is also one of the main organizations where HMs accumulated in plants^[37]^. When HM ions in the soil enter the root cells, they are mainly combined with certain components of the cell walls, such as cellulose, lignin, and pectin^[38]^. Under HM stresses, lignin as a metal absorption matrix changes in composition, deposition and content to adapt HM environment^[39]^. Aluminium (Al) toxicity in acidic soil is one of the principal factors that inhibit plant growth, especially root growth^[40]^, which is related to the accumulation of lignin in the cell wall. In *Pinus sylvestris* L.^[41]^ and *Melaleuca bracteata*^[40]^, excessive Al leads to increased lignin formation in the root system (Table 1). Tea (*Camellia sinensis* L.) is a perennial acidophilic woody plant and Al hyperaccumulator, and its growth is significantly stimulated by soil available Al^[42]^. Under 400 µM AlCl_3_ stress conditions, the activities of PAL and POD (PRX) in the cell wall decreased, and led to a decrease in lignin content^[43]^. Through comparative proteomic approaches, it was found that the down-regulation of *F5H* and the binding of cell wall phenolic acids to Al could reduce the accumulation of lignin in the roots of tea plants under Al stress (Table 1, Fig. 1)^[44]^. This might be the reason for the enhanced growth of tea plants under Al stress. In addition, Al stress can induce the expression of lignin biosynthetic genes in some woody plants. In *Citrus sinensis* and *Citrus* *grandis*, 1.0 mM AlCl_3_ treatment for 18 weeks induced the expression of *ALS3* (Al sensitive 3) and *CAD*, and increased the lignin content in roots (Table 1, Fig. 1)^[45]^.

#### Cadmium (Cd)

Similar to Al, cadmium (Cd) stress also induces lignin biosynthesis in different plant tissues, such as* C. sinensis* root and stem callus^[46]^, and *Avicennia schaueriana* xylem cells in all vegetative organs^[47]^. Cd exposure can increase the lignin content in poplar stems and roots^[48]^, and *Pinus sylvestris* roots^[49]^ by stimulating the activity of POD, a key enzyme in the process of lignin polymerization (Table 1). The Casparian strip is a specialized paracellular structure deposited in the root endodermis, consisting of a ring-like impregnation of the cell wall with lignin^[50]^. Casparian strips have been reported to limit Cd uptake by the roots in herbaceous hyperaccumulators such as *Sedum alfredii*^[51]^. For woody plants, the willow species is a cadmium-tolerant woody plant and is usually used to stabilize and reclaim soil contaminated by Cd^[52]^. In *Salix caprea* 'KH21' collected from an old mining area, the Casparian strip was farther to the root apex than the genetically distinct 'F20' isolate from a non-polluted area, which is an adaptive response to Cd stress. However, the Cd accumulation in leaves was higher in 'KH21' than in 'F20'. The possible reason is that 'KH21' delayed the development of the apoplastic barriers upon exposure to Cd (Table 1)^[53]^. In addition, in the root of *Aegiceras corniculatum* L. Blanco, the Casparian strip of the endodermis is considered to be the main factor contributing to Cd tolerance, and its Casparian strip takes part in the 'retardation mechanism' (Table 1)^[54]^. Therefore, similar to the role of lignin, Casparian strips as effective barriers protect the protoplast against the entry of Cd.

#### Copper (Cu) and zinc (Zn)

In addition to Al and Cd, the essential micronutrients copper (Cu) and zinc (Zn) play various roles in the normal growth and development of plants. In herbaceous plants such as *Matricaria chamomilla*^[55]^, *O. sativa*^[56]^ and *A. thaliana*^[57]^, excessive Cu or Zn can cause a significant increase in lignin content. In rice roots, Cu exposure significantly increased the expression of lignin biosynthetic genes (*OsCCoAOMT1* and *OsCCoAOMT20*) or lignin polymerization enzyme activities (POD/PRX and LAC) by Cu exposure (Fig. 1)^[56,58]^, and lignin accumulation increased with increasing concentrations and durations of Cu treatment^[58]^. Compared with herbaceous plants, there is little information about lignin deposition in woody plants in response to Cu or Zn stress. Excessive Cu or Zn increased the lignification in mangroves' roots, thereby preventing excessive Cu/Zn from further entering the roots^[59,60]^. Among the six species of mangrove seedlings, three species of highly metal-tolerant rhizophoraceous exhibited thick exodermis with high lignification and suberization (Table 1, Fig. 1). Besides, the tolerance index of mangroves is positively correlated with the deposition of lignin and suberin under Cu and Zn treatments (Table 1)^[61]^. These results indicate that enhanced lignification might be an adaptive strategy of woody plants to toxic HM ions.

### Other stresses – light and high CO_2_

#### Light

In addition to the above-mentioned stresses, other stresses such as shading stress also affect lignin biosynthesis. In the process of plant growth and development, shading stress seriously affected the photosynthesis and nutrient transport of plants, so the plants modified the overall architecture to intercept as much light as possible and enhanced the mechanical strength of the stem to minimize the lodging. In beech trees (*Fagus sylvatica* L.), the lignin content of leaves in the shade canopy is higher than in the sun canopy (Table 1)^[62]^. In maize-soybean intercropping systems, the lignin content also directly affects the lodging resistance of the stem. The shade-tolerant soybean cultivar 'Nandou 12' has a larger xylem area, and the accumulation of lignin and the activities of CAD, 4CL, PAL, and POD are higher than shade-susceptible cultivar 'Nandou 032-4' and the moderate shade-tolerant cultivar 'Jiuyuehuang' in intercropping^[63]^. Furthermore, photoperiod and light quality also affect the biosynthesis and deposition of lignin. When the tracheary elements differentiated from the cultured calli of *Pinus radiata*, the activity of CAD and PAL enzymes increased, and the content of lignin increased in the cell cultures grown from the dark to the 16 h photoperiod (Table 1, Fig. 1)^[64]^. In 'Fuji' apple (*Malus domestica* Borkh.) fruits, the expression levels of phenylpropanoid-related genes including *MdPAL*, chalcone synthase (*MdCHS*) and *MdF3H* were upregulated in the skin and flesh after sudden sunlight exposure for 29 days. However, the expression levels of* MdCOMT1* and *MdCAD* were highly induced in the flesh of 'Fuji' and 'Royal Gala' cultivars. A significant accumulation of lignin in cell walls of the flesh and skin was found in the sudden exposure, particularly in sun-injured 'Fuji' apples after 29 days (Table 1, Fig. 1)^[65]^. These studies indicate that the accumulation of lignin is induced in response to light stress to adapt to environmental changes.

#### High CO_2 _concentration

As an important substrate for photosynthesis, the concentration of CO_2_ directly influences the efficiency of photosynthesis, and even affects the morphology of plants. Wang et al. analyzed the differentially expressed proteins in fleshy roots of carrots under different CO_2_ treatments, and identified that the protein abundance of two lignin biosynthesis pathway proteins DcC4H and DcPER (DcPRX) increased under elevated CO_2_ stress, and the content of lignin also increased correspondingly (Table 1, Fig. 1)^[66]^. In hybrid poplar (*P. tremula* × *alba* 'INRA 717-1-B4'), elevated CO_2_ leads to increased carbon supply and lignin biosynthesis in the lower and middle stems of young trees (Table 1)^[67]^. However, elevated CO_2_ decreases the lignin content in birch (*Betula pendula*) leaves^[68]^ and beech (*Fagus sylvatica*) seedlings (Table 1)^[69]^.

## Transcription factors in the lignin regulatory network involved in the abiotic stress response

**Figure 2:**
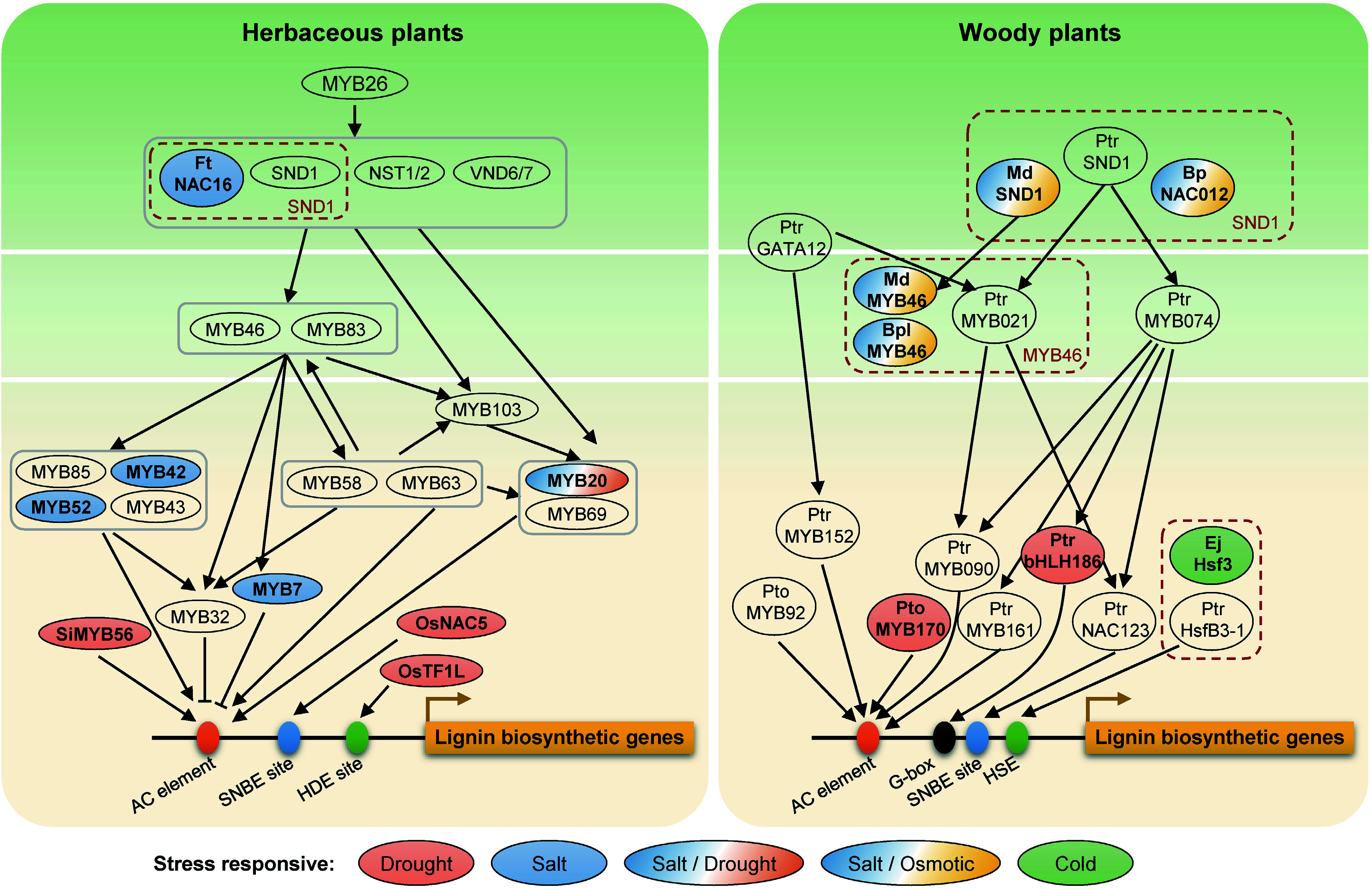
Transcription factors in the lignin regulatory network involved in abiotic stress responses in herbaceous and woody plants. Genes in red dashed boxes represent orthologous genes in different species. Different colors show different abiotic stresses. Ft, *Fagopyrum tataricum*; Si, *Setaria italic*; Os, *Oryza sativa*; Ptr, *Populus trichocarpa*; Pto, *Populus tomentosa*; Md, *Malus* × *domestica*; Bp or Bpl, *Betula platyphylla*; Ej, *Eriobotrya japonica*; No prefix, *Arabidopsis.* AC element, SNBE site, HDE site, G-box, and HSE located on the promoter region of lignin biosynthetic genes represent the *cis*-acting elements for the binding of MYB, NAC, TF1L, bHLH and HSF transcrition factors, respectively.

NAC domain transcription factors (SND1, NST1/2, VND6/7) as master switches in the first layer of the transcriptional regulatory network directly activate the expression of *MYB46* and *MYB83* in the second hierarchy^[70,71]^, which activate downstream transcription factors (including MYB20, MYB103, MYB42, MYB58 and MYB85) as the third hierarchy to activate lignin biosynthesis *via* regulating the structural genes (including *PAL1*, *PAL4*, *C3H*, *C4H*, *4CL1*,* 4CL2*, *4CL3*,* CCR*, *HCT*, *COMT*, *CCoAOMT*, *CAD6*, and *CAD9*) in *Arabidopsis*^[70]^. However, the SND1-mediated transcriptional regulatory network in a model tree species, *P. trichocarpa*, exhibited an obvious distinction compared to *Arabidopsis*^[72,73]^. In a four-layer *PtrSND1-B1*-mediated transcriptional regulatory network, *PtrSND1-B1* is the closest functional homolog of *AtSND1*. Poplar and Arabidopsis shared the same 'SND1-MYB46/83' regulatory module at the 1^st^−2^nd^ layers of the network ('*PtrSND1-B1-PtrMYB021*' module in poplar and '*AtSND1-AtMYB46/83*' in *Arabidopsis*)^[72]^. Whereas, only four common transcription factor orthologous were regulated by *PtrMYB021* and *AtMYB46* in the two species, respectively, suggesting that this regulatory network remains species-specific (Fig. 2). Interestingly, NAC and MYB transcription factors have a variety of important functions not only in plant growth and development but also in abiotic stress responses, such as drought, salt, and cold stresses^[74−76]^. Some stress-inducible NAC or MYB genes regulating lignin biosynthesis have been shown to be involved in abiotic stress tolerance.

### Master switches in the first and second layers – *SND1* and *MYB46*

Although the transcription factors regulating lignin biosynthesis-related genes have been extensively reported, whether these transcription factors are involved in abiotic stresses has not been thoroughly investigated. *FtNAC16* from tartary buckwheat (*Fagopyrum tataricum*), which is homologous to *AtNST1* and *AtNST3*, increased plant salt sensitivity by inhibiting S-lignin biosynthesis and reducing the total amount of lignin^[77]^. The orthologous gene of *SND1* in apple (*Malus* × *domestica* Borkh.), *MdSND1*, directly participates in the regulation of lignin biosynthesis by activating *MdMYB46/83* and in the signal transduction pathway in response to salt stress and osmotic stress through the stress-responsive transcription factors *MdAREB1A/1B*, *MdDREB2A*, *MdRD29A/22*, etc. (Table 1, Fig. 2)^[78]^. In white birch (*Betula platyphylla*), overexpression transcriptional activator *BpNAC012* (homolog of *SND1*) and *BplMYB46* directly activates the expression of lignin biosynthetic genes (*PAL*,* CCoAOMT*, *4CL*, *PRX*, and *LAC*) and other secondary cell wall-associated genes (*CESA*), and promotes the lignin deposition in secondary xylem and phloem fibers under salt and osmotic stresses (Table 1, Fig. 2)^[79,80]^. In *Arabidopsis*, *AtMYB20* regulated by *AtSND1* was developmentally associated with cells undergoing secondary wall thickening^[81]^. The expression of *AtMYB20* can be induced by high levels of NaCl, and overexpression of *MYB20* enhanced salt stress tolerance by negatively regulating the expression of PP2Cs, which is the major negative regulator of ABA signaling^[82]^. Meanwhile, *AtMYB20* was also pointed out to negatively regulate plant adaptive response to drought stress (Fig. 2)^[83]^. However, whether *AtMYB20* response to salt stress or drought stress is associated with lignin is unknown, and deserves further investigation. These results also indicated that the top layer of the lignin synthesis pathway responds to abiotic stresses and increases plant tolerance through the regulation of downstream genes.

### Third layer transcription factors

In* Populus* MYBs, *PtrMYB074* has been identified as one of the key wood formation regulators^[84]^, and the PtrMYB074-PtrWRKY19 dimers work combinatorially as an activator of *PtrbHLH186*, which is overexpressed in *P. trichocarpa* resulting in increased G-lignin, a higher proportion of smaller stem vessels and strong drought-tolerant phenotypes (Fig. 2)^[85]^. In *Arabidopsis*, the MPK4-mediated MYB42 phosphorylation enhanced the MYB42 transcriptional activity under salt-stress conditions, and then MYB42 directly bound to the* SALT OVERLY SENSITIVE 2* (*SOS2*) promoter and mediated the rapid induction of its expression after salt treatment^[86]^. Moreover, *AtMYB52* overexpression enhanced drought tolerance of *Arabidopsis* seedlings and salt-sensitivity by affecting the expression of genes involved in ABA metabolism or ABA response, which suggested a possible connection between cell wall biosynthesis and ABA-dependent growth regulation of seedlings^[87]^.

### Other transcription factors

In addition to transcription factors at the transcriptional regulatory network of lignin biosynthesis, other transcription factors also enhanced drought tolerance by regulating the functional genes in lignin biosynthesis. In rice, overexpression of the HD-Zip transcription factor *OsTF1L* (*transcription factor 1-like*) significantly improved the drought tolerance of transgenic plants during the vegetative growth stages and reduced the water loss rate^[88]^. Meanwhile, overexpressing* OsTF1L* up-regulated 29 lignin biosynthetic genes including *PRXs*, *CADs*, *COMTL5*, and *C4H* in shoots and promoted the lignin accumulation in leaves and stems^[88]^. Another transcription factor *OsNAC5* directly activates the expression of *OsCCR10*, and mediates drought tolerance by regulating lignin accumulation^[89]^. In addition, overexpressing *Setaria italic* *SiMYB56* in rice confers drought tolerance by up-regulating the expression of lignin biosynthesis genes *4CL5* and *F5H1* and ABA signaling pathway genes *P5CS* and *LEA7*^[90]^. In woody plants *P. tomentosa*,* PtoMYB170* promotes lignin deposition by directly activating the expression of *CCR2*, *CCoAOMT1*, *COMT2*, and *C4H2*, and can enhance the drought tolerance of transgenic plants (Table 1, Fig. 2)^[91]^.

Moreover, heat shock transcription factors (HSFs) regulated lignin biosynthesis under low temperature stress. The complex heat shock response network composed of HSFs and heat shock proteins (HSPs) is a major active protection mechanism under extreme temperatures^[92,93]^. In loquat (*Eriobotrya japonica*) fruit, different members of the HSF family play different regulatory roles in response to high/low temperatures and lignin biosynthesis. Under high temperature, *EjHSF1* is induced and subsequently activates the expression of downstream *HSPs* (*EjHSP70-2*, *EjsHSP1/4/5*, etc.); whereas under low temperature, *EjHSF3* is repressed and its direct activation of lignin biosynthetic genes (*EjPAL1*, *Ej4CL1/5*, etc.) is blocked by the accompanying EjHSF3-EjAP2-1 interaction (Table 1, Fig. 2)^[94]^.

## Conclusions and future perspective

As one of the main components of plant cell walls, lignin plays a vital role in plant growth and development and stress response. The biosynthesis of lignin is a complex biological process involving multiple enzymes, which is regulated by transcriptional and post-transcriptional regulations. Woody plants have to endure more abiotic stresses due to their perennial characteristics, and enhanced cell wall lignification, which not only provides mechanical support, but also provides a barrier against various abiotic stresses. For example, the plant-specific NAC transcription factor functions in vessel cell wall lignification and influences plant stress tolerance, such as salt, cold, and dehydration stress, by regulating downstream stress-responsive genes^[95]^. Similarly, in the model plant *Arabidopsis*, *VASCULAR-RELATED NAC-DOMAIN 7* (*VND7*) triggers xylem formation via promoting cell wall lignification after verticillium infection to enhance drought tolerance^[96]^. Moreover, blue light perceived by* Cryptochrome1* (*CRY1*) enhances secondary cell wall thickening in fiber cells via *MYC2/MYC4* activation of the *NST1*-directed transcriptional network^[97]^. In addition to transcriptional regulation, various abiotic stresses such as extreme temperature, waterlogging and drought, salinity, and HMs lead to changes in enzyme activity in the lignin biosynthesis pathway, thereby affecting the intermediate metabolites and lignin content (Fig. 1). However, the current understanding of the fine regulatory mechanism of lignin deposition in response to abiotic stresses is still very limited, which is mainly due to the complex genetic background and the limitations of genetic transformation of woody plants.

In recent years, the rapid development of molecular biology techniques and various omics analysis techniques have provided the possibility for in-depth analysis of forest lignin synthesis pathways and their regulatory mechanisms. For example, through the expression quantitative trait loci analysis based on a poplar natural population, a novel regulatory module PtWRKYs-PtHCT2 was successfully identified to participate in the biosynthesis of three metabolites in the lignin biosynthesis pathway and defense response^[98]^. In addition, several novel genes affecting lignin biosynthesis were successfully identified through multi-omics joint-analysis, e.g., serine hydroxymethyltransferase (*PdSHMT2*)^[99]^ and prefoldin chaperon (*PdPFD2.2*)^[100]^. Furthermore, the improvement of the accuracy of CRISPR/Cas-mediated gene editing has opened up a new field for further study of gene functions at the single-base level in woody plants. These new technologies and methods provide a powerful solution for expanding the application of lignin in tree breeding of abiotic stress resistance.
